# Wastewater-based surveillance data to determine the COVID-19 trends in communities with low population

**DOI:** 10.1016/j.dib.2025.111756

**Published:** 2025-06-07

**Authors:** Aiswarya Rani Pappu, Ashley Green, Melanie Oakes, Sunny Jiang

**Affiliations:** a Department of Civil and Environmental Engineering, University of California Irvine, Irvine, USA; b Department of Biological Chemistry, University of California Irvine, Irvine, USA

**Keywords:** Wastewater SARS-CoV-2, Wastewater PMMoV, GIS, Epidemiological trend analysis, Low population communities

## Abstract

The data presented in this article show SARS-CoV-2 viral concentration and trends in wastewater among communities with different population size. Particularly, the data show that wastewater SARS-CoV-2 concentration can better predict COVID-19 transmission in communities than clinical data. The article also reports PMMoV data in wastewater and population data in each community, and their effects on the correlation between wastewater SARS-CoV-2 and clinical COVID-19 data. The wastewater and clinical data reported in this article are collected from 7 students’ housing communities with population ranging between 300 and 4000 residents per community. The dataset presents SARS-CoV-2 N2 and E gene as well as PMMoV concentrations in the raw wastewater samples collected from 13 sewer manholes at these communities roughly three times per week for a period of 6-months between December 2021 and June 2022. This dataset will help to 1) improve future wastewater based epidemiological models, 2) improve understanding on PMMoV concentration ranges in wastewater at low population communities, 3) develop methods to support data interpretation, and 4) understand the effects of spatial scales on sampling frequency and infection outbreak detection.

Specifications Table

**Table:** 

Subject	Earth & Environmental Sciences
Specific subject area	SARS-CoV-2 and PMMoV concentrations in wastewater collected at low population communities during the COVID-19 pandemic
Type of data	Processed and analysed. Data provided in figures and tables.
Data collection	Data were collected from a wastewater sampling campaign conducted between December 2021 and June 2022. Wastewater samples were collected as 24-hour time-weighted composites from sewer manholes in students’ housing communities with populations ranging from 300 to 4000 people. Dataset shows SARS-CoV-2 N2 and E gene concentrations and Pepper Mild Mottle Virus (PMMoV) concentrations in 726 samples detected using RT-ddPCR in raw wastewater. COVID-19 clinical testing data were obtained from University of California Irvine for the span of the study period. Spearman correlation analysis and quantitative trend analysis data provided in this article were performed using RStudio®. Spatiotemporal visualization of infection hotspot clusters was performed using ArcGIS.
Data source location	University of California, Irvine (33.6405407712 -117.838914978), Irvine, California, USA
Data accessibility	Wastewater surveillance data are provided in Mendeley Data repository Repository name: Mendeley Data Data identification number: 10.17632/6x5zffpxwb.3 Direct URL to data: https://data.mendeley.com/datasets/6x5zffpxwb/3 Clinical data are provided in table and graph formats in this article.
Related research article	Pappu, A.R., Green, A., Oakes, M. and Jiang, S., 2025. Tracking COVID-19 trends in communities with low population by wastewater-based surveillance. Science of The Total Environment, 970, p.179007. 10.1016/j.scitotenv.2025.179007

## 1 Value of the Data

• This dataset provides insights into wastewater SARS-CoV-2 and PMMoV concentration ranges at low population communities. This is especially useful for epidemiological studies.
• This dataset provides insights on changing correlations between wastewater pathogen data and clinical cases during relaxation of COVID-19 restrictions and changing public testing behavior.
• The data on virus prevalence and concentration ranges in wastewater at various population sizes are valuable for researchers in improving wastewater-based epidemiological models.
• Insights derived from this data can aid researchers in enhancing future epidemiological models with public behavioral factors.

## 2 Background

This study collects wastewater SARS-CoV-2 and clinical case surveillance data to understand their ability to predict COVID-19 infection trends during the relaxation period of COVID-19 regulations. Wastewater was collected from 13 sewer manholes serving 7 communities with population size ranging between 300 and 4000 residents for six months. The SAR-CoV-2 N2 and E gene and PMMoV concentration were measured in the 726 wastewater samples. The wastewater data were analyzed for association with clinical COVID-19 cases. And a quantitative trend analysis was conducted to estimate wastewater’s ability to detect changes in community infection trends when formal clinical testing was on the decline.

## 3 Data Description

**Fig. 1:**
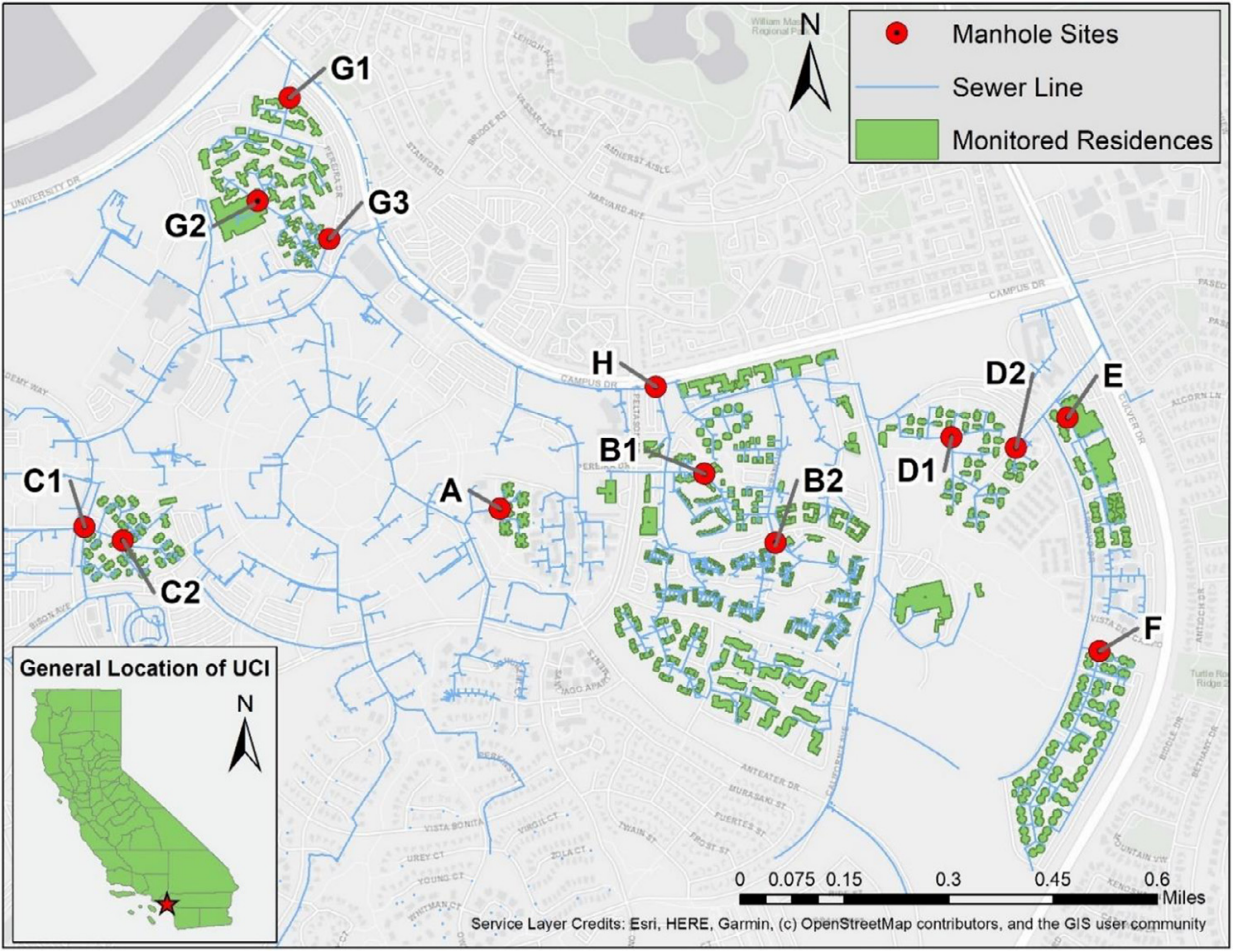
Map of University of California, Irvine main campus showing buildings (gray boxes) and underground sewer lines (blue lines). Red dots mark the sewer manholes sampled in this study. The student dorm buildings that connected with sampled manholes are indicated by green polygons. The student communities are labeled from A to H according to the population size from the smallest to the largest. One to three manholes were collected within each community. (For interpretation of the references to color in this figure legend, the reader is referred to the web version of this article.).

This dataset was collected at the University of California, Irvine (Irvine, California) from December 2021 to June 2022. Wastewater samples were taken from seven student residential housing communities (A to G) and one confluent site (H) (Fig. 1). The population size among the monitored communities ranges from 300 to 4000 people. Raw wastewater samples were collected from 13 sewer manholes located on gravity sewer lines, which do not capture any stormwater runoff. Between one to three sewer manholes were monitored at each community (Table 1). In total, 726 wastewater samples were collected and analyzed during the study period.

**Table 1:** Summary of communities monitored through wastewater surveillance project on the University of California, Irvine main campus.

Community Designation	Buildings Monitored	Population Monitored	Number of manholes monitored	Number of samples collected and tested/sampling events performed (a)	% Sampling failure
A	6	300	1	47/65	30.7 %
B	17	464	2 (B1, B2)	97/130 (47, 50)	25.3 %
C	32	608	2 (C1, C2)	98/130 (37, 61)	24.6 %
D	34	798	2 (D1, D2)	124/130 (60, 64)	4.6 %
E	11	1089	1	64/65	1.5 %
F	21	1236	1	62/65	4.6 %
G	30	2392	3 (G1, G2, G3)	170/195 (56, 50, 64)	12.8 %
H	111	3970	1	64/65	1.5 %

a values in parenthesis represents total samples collected at each manhole.

Wastewater collected from the monitored communities was analysed for SARS-CoV-2 and PMMoV concentrations. All samples collected during this study (Table 1) were analyzed and no samples were excluded during the sample analysis. The data and analysis presented in tables and figures are supplemental to the accompanying article [4]. The data of percent positive samples among the total collected samples, and average, standard deviation and range (minimum and maximum) of SARS-CoV-2 N2 gene and PMMoV concentrations detected in wastewater samples at each community are shown in Table 2. Analyses of SARS-CoV-2 E gene in relationship to N gene are presented in [6].

**Table 2:** Summary of wastewater SARS-CoV-2 N2 gene and PMMoV concentrations detected in wastewater samples.

Community	SARS-CoV-2 N2 gene Concentration			PMMoV Concentration		
	% Positive samples	Mean (Std. dev.) GC/mL	Range GC/mL (Min. – Max.)	% Positive samples	Mean (Std. dev.) GC/mL	Range GC/mL (Min. – Max.)
A	36.1 %	4.45 × 10^2^ (1.30 × 10^3^)	ND to 7.48 × 10^3^	100 %	1.67 × 10^6^ (4.42 × 10^6^)	6.22 × 10^4^ to 2.96 × 10^7^
B	41.2 %	5.63 × 10^2^ (1.89 × 10^3^)	ND to 1.18 × 10^4^	98.9 %	5.29 × 10^6^ (1.86 × 10^7^)	7.49 × 10^4^ to 1.42 × 10^8^
C	33.6 %	3.92 × 10^2^ (1.67 × 10^3^)	ND to 1.28 × 10^4^	100 %	6.74 × 10^6^ (2.96 × 10^7^)	5.02 × 10^4^ to 2.05 × 10^8^
D	32.2 %	4.07 × 10^2^ (2.02 × 10^3^)	ND to 1.61 × 10^4^	100 %	2.35 × 10^6^ (6.19 × 10^6^)	4.66 × 10^4^ to 4.14 × 10^7^
E	51.5 %	5.43 × 10^2^ (1.23 × 10^3^)	ND to 6.16 × 10^3^	100 %	1.87 × 10^7^ (1.39 × 10^8^)	9.10 × 10^3^ to 1.11 × 10^9^
F	41.9 %	3.12 × 10^2^ (8.59 × 10^2^)	ND to 5.48 × 10^3^	98.3 %	9.88 × 10^5^ (1.35 × 10^6^)	3.87 × 10^4^ to 6.96 × 10^6^
G	43.5 %	6.76 × 10^2^ (1.99 × 10^3^)	ND to 1.37 × 10^4^	100 %	1.69 × 10^6^ (3.29 × 10^6^)	1.12 × 10^5^ to 2.53 × 10^7^
H	53.1 %	7.74 × 10^2^ (2.30 × 10^3^)	ND to 1.33 × 10^4^	100 %	1.40 × 10^6^ (1.08 × 10^6^)	4.00 × 10^4^ to 5.69 × 10^6^
Agg	98.5 %	6.04 × 10^2^ (1.31 × 10^3^)	ND to 7.10 × 10^3^	100 %	3.30 × 10^6^ (1.16 × 10^7^)	1.77 × 10^5^ to 9.34 × 10^7^

*Note:* GC/mL represents genome copies per millilitre of wastewater sample. ND shows that the wastewater virus concentrations were below the detection limit. ‘Agg’ represents the aggregated wastewater SARS-CoV-2 concentration from all monitored communities.

**Fig. 2:**
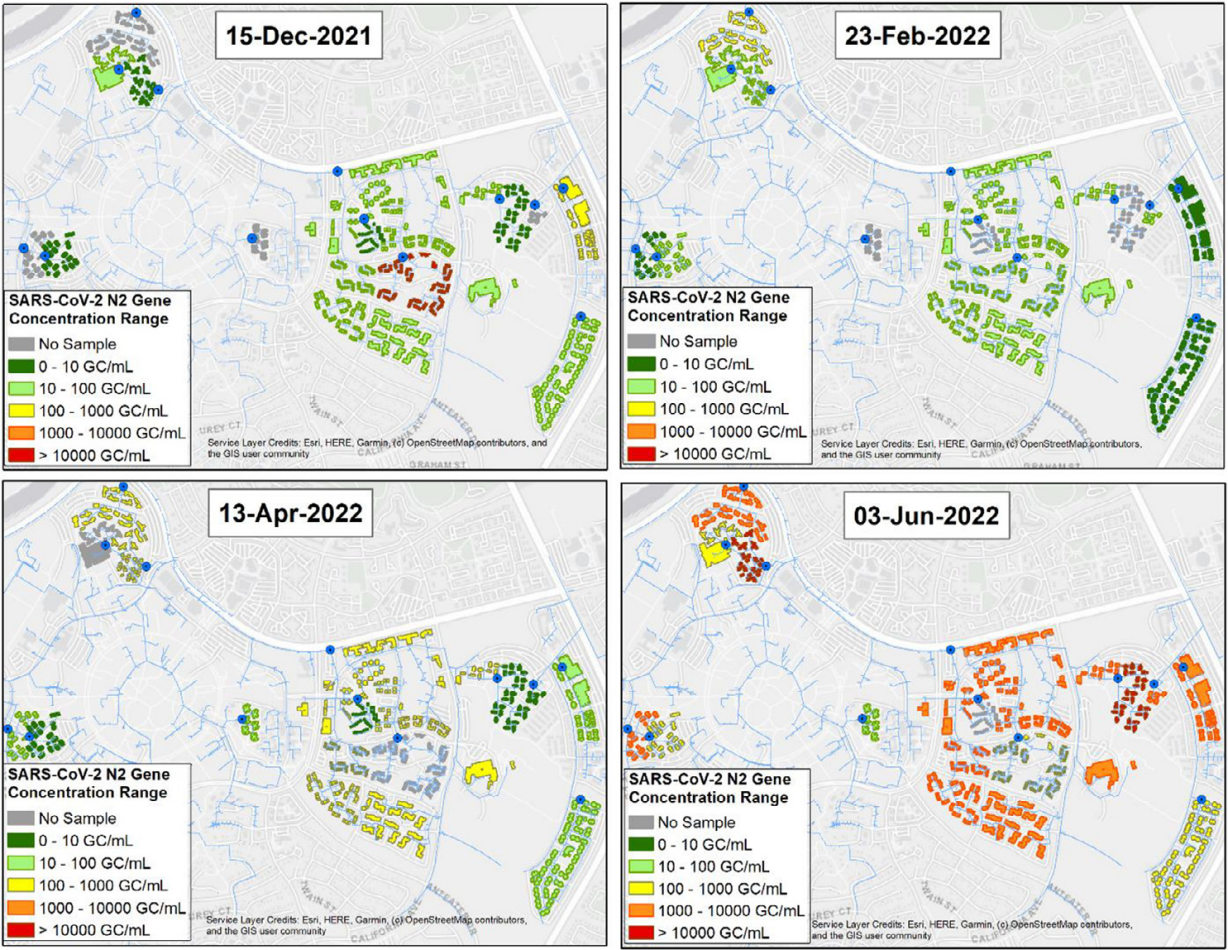
Spatial-temporal visualization of wastewater SARS-CoV-2 RNA concentration in the monitored student housing communities using ArcGIS. The color gradient represents the range of SARS-CoV-2 N2 gene concentration (GC/mL) in wastewater, light blue lines represent sewer line network, and blue circles represent sewer manhole locations. (For interpretation of the references to color in this figure legend, the reader is referred to the web version of this article.).

**Fig. 3:**
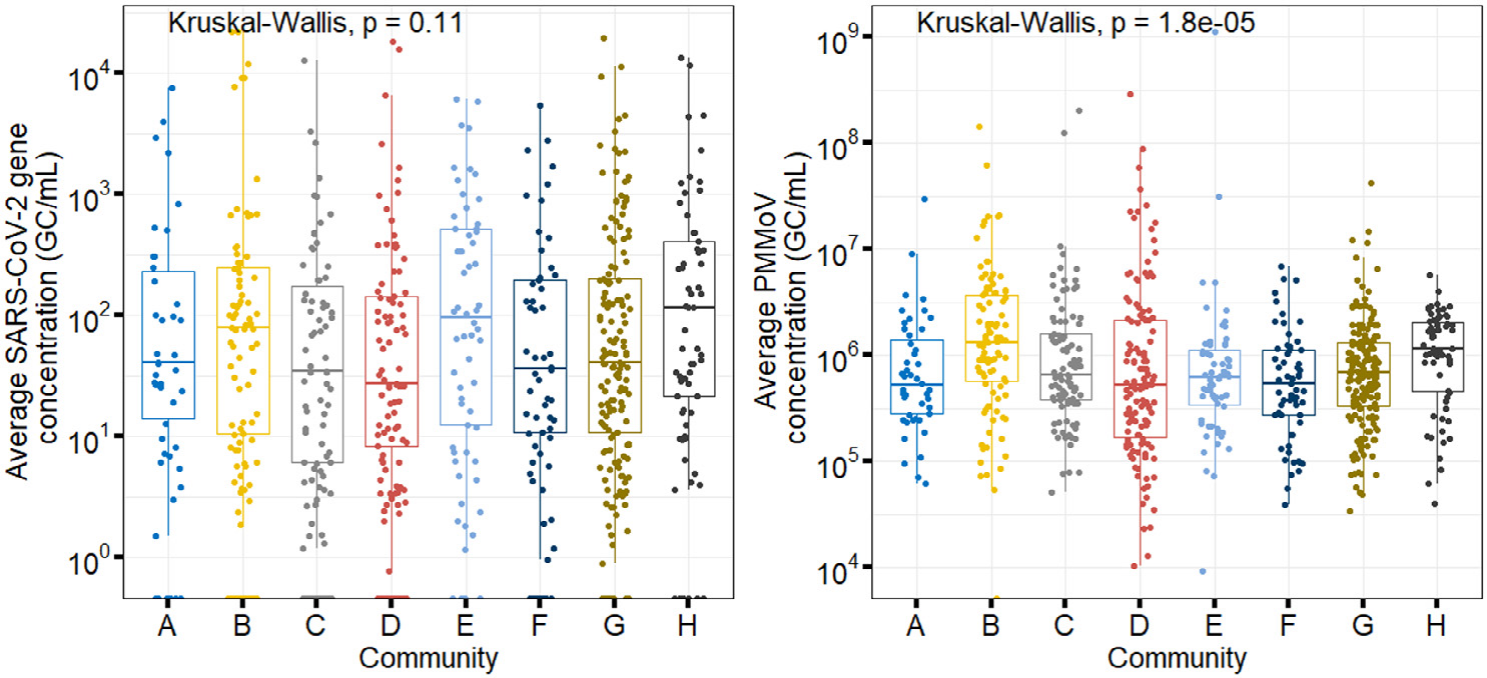
Distribution of average SARS-CoV-2 N2 gene (left) and PMMoV (right) concentrations detected in wastewater samples by monitored community. Sites are ordered based on increasing population size.

Fig. 2 provides ArcGIS maps showing infection hotspot cluster(s) identified on certain days of the study period, while Fig. 3 shows the distribution of average SARS-CoV-2 N2 gene and PMMoV concentration observed at each community during the 6-month sampling period. Kruskal-Wallis test values reveal the statistical differences between the average virus concentrations detected in wastewater. The average concentration of wastewater SARS-CoV-2 N2 gene were used for spatio-temporal visualization of infection hotspot clusters among the monitored communities using ArcGIS.

The SARS-CoV-2 N2 data were evaluated together with clinical COVID-19 cases to assess their correlations in communities with varying population size. For this purpose, Spearman correlation analysis was conducted between wastewater SARS-CoV-2 and COVID-19 clinical cases. Results reported in Table 3 shows correlations of wastewater SARS-CoV-2 with clinical data for various scenarios of normalization (without normalization, normalization by PMMoV, and normalization by population size), and with and without averaging of clinical case data (daily values, 3-day rolling average, and 7-day rolling average) for each community. The absolute value of spearman rho (|ρ|) correlation coefficient values between 0 to 0.19, 0.2 to 0.39, 0.4 to 0.59, 0.6 to 0.79, and 0.8 to 1 were considered very weak, weak, average, strong and very strong correlations, respectively.

**Table 3:** Spearman correlation of wastewater SARS-CoV-2 N2 (with and without normalized by PMMoV or by population size) with COVID-19 clinical cases (daily (CC), 3-day rolling average (CC-3D), and 7-day rolling average (CC-7D)) for each community.

Community	N	Raw wastewater SARS-CoV-2 N2 gene concentration			PMMoV Normalized wastewater SARS-CoV-2 N2 gene concentration			Population Normalized wastewater SARS-CoV-2 N2 gene concentration		
		CC	CC-3D	CC-7D	CC	CC-3D	CC-7D	CC-7D	CC-3D	CC-7D
A	47	0.34	0.39	0.38	**0.27^ns^**	0.36	0.43	0.36	0.40	0.39
B	62	0.53	0.61	0.58	0.46	0.56	0.57	0.55	0.60	0.56
C	62	0.10^ns*^	0.29	**0.14^ns^**	**0.06^ns^**	0.37	**0.27^ns^**	**0.10^ns^**	0.31	**0.17^ns^**
D	63	**0.24^ns^**	0.25	0.42	**0.19^ns^**	**0.16^ns^**	0.36	0.28	0.28	0.43
E	62	0.40	0.43	0.47	0.49	0.52	0.52	0.40	0.44	0.48
F	60	0.30	0.41	0.51	0.31	0.38	0.58	0.32	0.42	0.53
G	63	0.52	0.62	0.59	0.53	0.68	0.70	0.55	0.65	0.65
H	62	0.57	0.61	0.59	0.59	0.69	0.73	0.58	0.63	0.61
Agg	63	0.55	0.62	0.63	0.56	0.70	0.75	0.58	0.64	0.66

*Note:* ‘ns’ represents non-significant values. COVID-19 daily clinical cases, 3-day rolling average of clinical cases, and 7-day rolling average of clinical cases are represented as CC, CC-3D, and CC-7D, respectively.

**Fig. 4:**
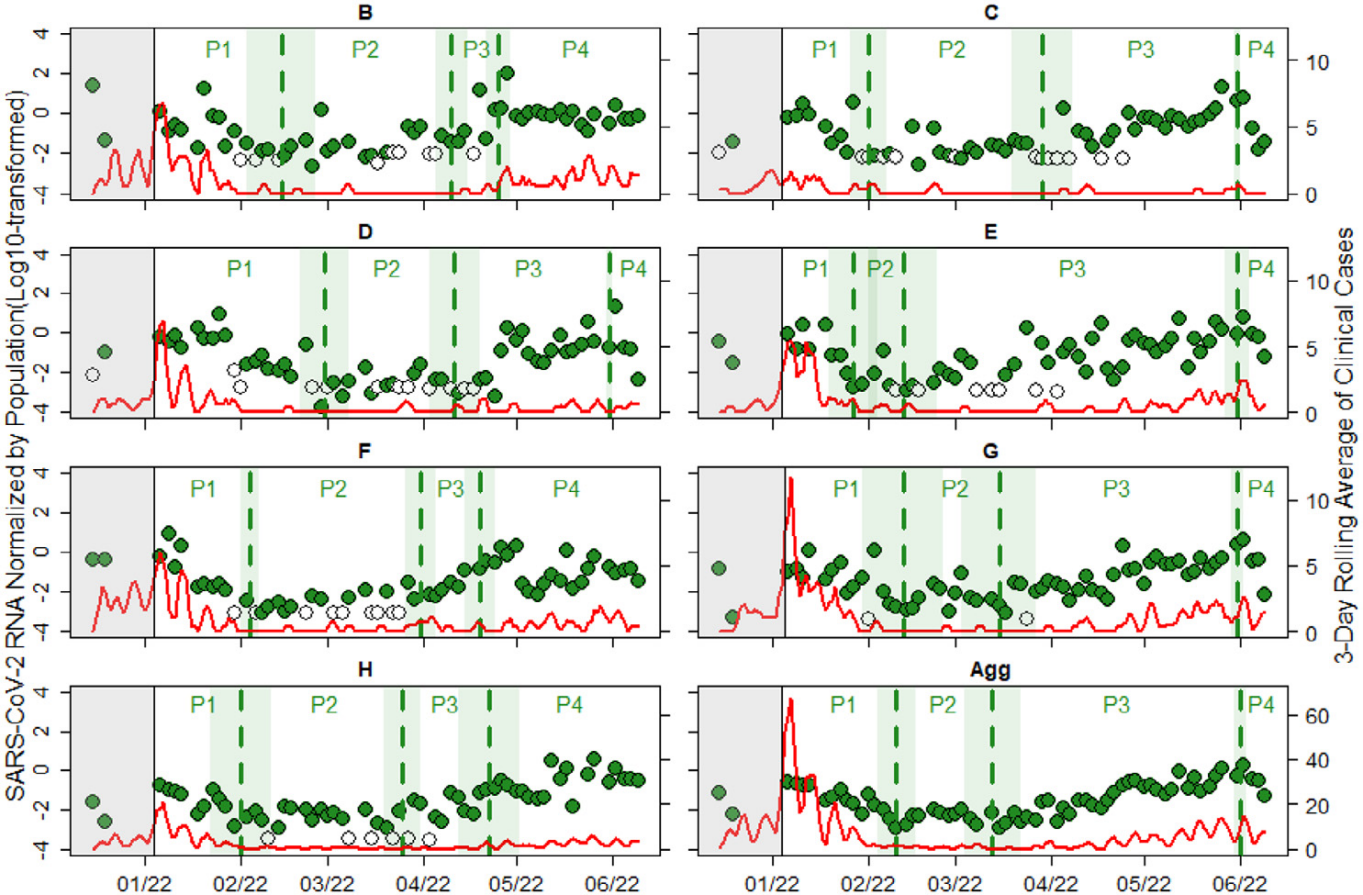
Longitudinal plot of log-transformed SARS-CoV-2 N2 gene data (green) that normalized by the community population size overlaid with 3-day rolling average of clinical reported cases (red line) from individual community and aggregated data from all UCI communities. Green vertical dash lines indicate the breakpoints estimated through SLR, representing potential dates of trend changes in wastewater SARS-CoV-2 N2 gene data. The left gray region shows data not included in the SLR. The green shaded region represents the standard error of the breakpoint. P1, P2, P3, and P4 represent the phases (P#) of major changes in wastewater SARS-CoV-2 N2 gene data. Hollow points show imputed wastewater data.(For interpretation of the references to color in this figure legend, the reader is referred to the web version of this article.).

A quantitative trend analysis was conducted to estimate the ability of wastewater and clinical surveillance to determine infection incidence under changing public testing behavior. For this purpose, the dates and phases of major trend changes in wastewater SARS-CoV-2 data were estimated using segmented linear regression. Table 4 shows the results of breakpoint (BP) dates and respective standard error (in days) estimated from segmented linear regression analysis of LOESS (locally estimated scatterplot smoothing) fit log transformed population normalized wastewater SARS-CoV-2 data. Breakpoint(s) represents the date(s) when a major trend change in wastewater virus data has occurred. Table 5 shows comparison of summary statistics of observed data and population normalized wastewater SARS-CoV-2 concentration data imputed using LOESS fit smoothing factor of 0.04. Fig. 4 shows a longitudinal plot of log-transformed SARS-CoV-2 N2 data that was normalized by the community population size overlaid with 3-day rolling average of clinical reported cases from individual communities and the aggregated data from all communities. It also provides visual representation of the BPs, standard errors of the BPs, and segments of phases (P#) of major changes in wastewater SARS-CoV-2 trends estimated using segmented linear regression. Mann Kendall (MK) tau and daily percent change (PC) trend analysis were performed on the estimated segments to interpret the direction and magnitude of trend change in wastewater SARS-CoV-2 N2 gene and clinical case data. Tables 6 and 7 show summaries of MK and PC results of 3-day rolling average of COVID-19 clinical case trends and population normalized wastewater SARS-CoV-2 trends.

**Table 4:** Summary of breakpoint (BP) date and standard error (in days) estimates from segmented linear regression analysis of population normalized wastewater SARS-CoV-2 trends.

Community	Breakpoint ID	Breakpoint Date	Breakpoint Std Err. (days)
B	BP1	2/14/2022	12
	BP2	4/10/2022	5
	BP3	4/25/2022	5
C	BP1	2/1/2022	6
	BP2	3/29/2022	10
	BP3	5/31/2022	2
D	BP1	2/28/2022	8
	BP2	4/11/2022	8
	BP3	5/31/2022	2
E	BP1	1/27/2022	9
	BP2	2/12/2022	11
	BP3	5/31/2022	5
F	BP1	2/4/2022	4
	BP2	3/31/2022	6
	BP3	4/19/2022	5
G	BP1	2/12/2022	13
	BP2	3/15/2022	13
	BP3	5/31/2022	3
H	BP1	2/1/2022	11
	BP2	3/25/2022	6
	BP3	4/22/2022	10
Agg	BP1	2/10/2022	6
	BP2	3/13/2022	10
	BP3	6/1/2022	2

*Note:* Community A data was not analyzed due to large data gaps; BP1 represents the transition from P1 to P2 (declined to plateau), BP2 represents the transition from P2 to P3 (plateau to escalation), and BP3 represents the transition from P3 to P4 (escalation to decline or plateau).

**Table 5:** Summary statistics of observed data and imputed population normalized wastewater SARS-CoV-2 N2 gene concentration data.

Community	Observed			Imputed		
	N	Mean ±SD	Min to max (GC/mL)	N	Mean ±SD	Min to max (GC/mL)
B	62	2.64 ± 0.14	2.29 × 10^-3^ to 1.08 × 10^2^	155	2.58 ± 0.12	2.29 × 10^-3^ to 1.08 × 10^2^
C	62	0.84 ± 2.95	2.77 × 10^-3^ to 2.21 × 10^1^	155	0.99 ± 3.08	2.77 × 10^-3^ to 2.21 × 10^1^
D	63	0.73 ± 2.93	1.75 × 10^-4^ to 2.18 × 10^1^	155	0.71 ± 2.50	1.75 × 10^-4^ to 2.18 × 10^1^
E	61	0.59 ± 1.23	1.02 × 10^-3^ to 5.85	155	0.59 ± 1.06	1.02 × 10^-3^ to 5.85
F	59	0.34 ± 1.21	8.03 × 10^-4^ to 8.87	155	0.33 ± 1.03	8.03 × 10^-4^ to 8.87
G	63	0.27 ± 0.71	4.19 × 10^-4^ to 4.57	155	0.30 ± 0.67	4.19 × 10^-4^ to 4.57
H	61	0.22 ± 0.63	2.9 × 10^-4^ to 3.61	155	0.24 ± 0.59	2.9 × 10^-4^ to 3.61

**Table 6:** Summary of Mann Kendall Tau results of 3-day rolling average of COVID-19 clinical case trends and population normalized wastewater SARS-CoV-2 N2 gene trends.

Community	Window	Window Duration	3-Day rolling average of clinical case trends	Population normalized wastewater SARS-CoV-2 trends
			MK^(a)^	
B	P1	01/06/22 - 02/14/22	−0.667 (*p* < 0.001)	−0.344 (*p* < 0.01)
	P2	02/14/22 - 04/10/22	−0.257 (*p* < 0.05)	**0.115 (ns)**
	P3	04/10/22 - 04/25/22	**0.362 (ns)**	0.429 (*p* < 0.05)
	P4	04/25/22 - 06/09/22	**0.113 (ns)**	−0.299 (*p* < 0.01)
C	P1	01/06/22 - 02/01/22	−0.394 (*p* < 0.01)	−0.339 (*p* < 0.05)
	P2	02/01/22 - 03/29/22	−0.306 (*p* < 0.01)	**0.079 (ns)**
	P3	03/29/22 - 05/31/22	**0.071 (ns)**	0.544 (*p* < 0.001)
	P4	05/31/22 - 06/09/22	−0.645 (*p* < 0.05)	−0.778 (*p* < 0.01)
D	P1	01/06/22 - 02/28/22	−0.63 (*p* < 0.001)	−0.529 (*p* < 0.001)
	P2	02/28/22 - 04/11/22	0.267 (*p* < 0.05)	**0.067 (ns)**
	P3	04/11/22 - 05/31/22	**0.141 (ns)**	0.428 (*p* < 0.001)
	P4	05/31/22 - 06/09/22	0.704 (*p* < 0.05)	−0.722 (*p* < 0.01)
E	P1	01/06/22 - 01/27/22	−0.63 (*p* < 0.001)	−0.472 (*p* < 0.01)
	P2	01/27/22 - 02/12/22	**0.029 (ns)**	−**0.167 (ns)**
	P3	02/12/22 - 05/31/22	0.484 (*p* < 0.001)	0.508 (*p* < 0.001)
	P4	05/31/22 - 06/09/22	−0.761 (*p* < 0.01)	−0.889 (*p* < 0.01)
F	P1	01/06/22 - 02/04/22	−0.602 (*p* < 0.001)	−0.683 (*p* < 0.001)
	P2	02/04/22 - 03/31/22	0.321 (*p* < 0.01)	0.301 (*p* < 0.01)
	P3	03/31/22 - 04/19/22	−**0.331 (ns)**	0.86 (*p* < 0.001)
	P4	04/19/22 - 06/09/22	0.316 (*p* < 0.01)	−**0.095 (ns)**
G	P1	01/06/22 - 02/12/22	−0.82 (*p* < 0.001)	−0.451 (*p* < 0.001)
	P2	02/12/22 - 03/15/22	**0.159 (ns)**	**0.024 (ns)**
	P3	03/15/22 - 05/31/22	0.615 (*p* < 0.001)	0.558 (*p* < 0.001)
	P4	05/31/22 - 06/09/22	−**0.254 (ns)**	−0.778 (*p* < 0.01)
H	P1	01/06/22 - 02/01/22	−0.656 (*p* < 0.001)	−0.573 (*p* < 0.001)
	P2	02/01/22 - 03/25/22	−**0.159 (ns)**	−0.24 (*p* < 0.05)
	P3	03/25/22 - 04/22/22	**0.178 (ns)**	0.392 (*p* < 0.01)
	P4	04/22/22 - 06/09/22	0.318 (*p* < 0.01)	0.225 (*p* < 0.05)
Agg	P1	01/06/22 - 02/10/22	−0.745 (*p* < 0.001)	−0.514 (*p* < 0.001)
	P2	02/10/22 - 03/13/22	−**0.069 (ns)**	**0.114 (ns)**
	P3	03/13/22 - 06/01/22	0.666 (*p* < 0.001)	0.686 (*p* < 0.001)
	P4	06/01/22 - 06/09/22	−**0.286 (ns)**	−0.857 (*p* < 0.01)

*Note:* ‘ns’ stands for non-significant. (a) values in parenthesis represent p-value of Mann Kendall test. Bolded values represent non-significant values.

**Table 7:** Summary of daily percent change (PC) results of 3-day rolling average of COVID-19 clinical case trends and population normalized wastewater SARS-CoV-2 N2 gene trends.

Community	Window	Window Duration	3-Day rolling average of clinical case trends	Population normalized wastewater SARS-CoV-2 trends
			PC^(a)^	
B	P1	01/06/22 - 02/14/22	−22.94 (*p* < 0.001)	−7.08 (*p* < 0.01)
	P2	02/14/22 - 04/10/22	−0.6 (*p* < 0.05)	**0.73 (ns)**
	P3	04/10/22 - 04/25/22	**6.36 (ns)**	35.34 (*p* < 0.05)
	P4	04/25/22 - 06/09/22	**2.11 (ns)**	−5.24 (*p* < 0.001)
C	P1	01/06/22 - 02/01/22	−8.13 (*p* < 0.01)	−12.05 (*p* < 0.01)
	P2	02/01/22 - 03/29/22	−0.99 (*p* < 0.01)	−**0.34 (ns)**
	P3	03/29/22 - 05/31/22	**0.22 (ns)**	9.68 (*p* < 0.001)
	P4	05/31/22 - 06/09/22	−11.63 (*p* < 0.05)	−56.79 (*p* < 0.001)
D	P1	01/06/22 - 02/28/22	−13.17 (*p* < 0.001)	−10.42 (*p* < 0.001)
	P2	02/28/22 - 04/11/22	**1.1 (ns)**	**0.77 (ns)**
	P3	04/11/22 - 05/31/22	**0.76 (ns)**	12.44 (*p* < 0.001)
	P4	05/31/22 - 06/09/22	18.09 (*p* < 0.01)	−64.38 (*p* < 0.001)
E	P1	01/06/22 - 01/27/22	−42.28 (*p* < 0.001)	−23.05 (*p* < 0.001)
	P2	01/27/22 - 02/12/22	−**0.17 (ns)**	−**1.1 (ns)**
	P3	02/12/22 - 05/31/22	2.29 (*p* < 0.001)	6.06 (*p* < 0.001)
	P4	05/31/22 - 06/09/22	−50.83 (*p* < 0.001)	−38.68 (*p* < 0.001)
F	P1	01/06/22 - 02/04/22	−28.44 (*p* < 0.001)	−21.78 (*p* < 0.001)
	P2	02/04/22 - 03/31/22	1.3 (*p* < 0.01)	3.22 (*p* < 0.001)
	P3	03/31/22 - 04/19/22	−6.51 (*p* < 0.05)	23.63 (*p* < 0.001)
	P4	04/19/22 - 06/09/22	3.43 (*p* < 0.01)	−**2.24 (ns)**
G	P1	01/06/22 - 02/12/22	−35.54 (*p* < 0.001)	−9.59 (*p* < 0.001)
	P2	02/12/22 - 03/15/22	**0.65 (ns)**	**0.45 (ns)**
	P3	03/15/22 - 05/31/22	5.63 (*p* < 0.001)	7.33 (*p* < 0.001)
	P4	05/31/22 - 06/09/22	−**32.31 (ns)**	−48.01 (*p* < 0.01)
H	P1	01/06/22 - 02/01/22	−70.22 (*p* < 0.001)	−10.58 (*p* < 0.001)
	P2	02/01/22 - 03/25/22	−**0.94 (ns)**	−**1.88 (ns)**
	P3	03/25/22 - 04/22/22	9.94 (*p* < 0.05)	11.07 (*p* < 0.01)
	P4	04/22/22 - 06/09/22	10.69 (*p* < 0.001)	4.62 (*p* < 0.01)
Agg	P1	01/06/22 - 02/10/22	−94.59 (*p* < 0.001)	−7.4 (*p* < 0.001)
	P2	02/10/22 - 03/13/22	−**0.34 (ns)**	**2.03 (ns)**
	P3	03/13/22 - 06/01/22	33.89 (*p* < 0.001)	7.12 (*p* < 0.001)
	P4	06/01/22 - 06/09/22	−**89.04 (ns)**	−29.79 (*p* < 0.001)

*Note:* ‘ns’ stands for ‘non-significant’. (a) values in parenthesis represent p-value of percent change analysis. Bolded values represent non-significant values.

## 4 Experimental Design, Materials and Methods

Wastewater samples were collected from the sewer manholes three times per week (on Monday, Wednesday, and Friday) using a HACH AS950 autosampler (Hach, Loveland, CO, United States). Autosampler was programmed to collect 24-h time weighted wastewater composite samples at 1-h interval. Samples were drawn up from bottom of the manhole through a perforated metal nozzle to ensure immersion of intake port in the wastewater flow and reduce clogging by debris in wastewater. Collected wastewater samples were homogenized and aliquoted into 50 mL centrifuge tubes and were transported to lab on ice for SARS-CoV-2 and PMMoV quantification on the same day. A chain of custody was filled during each sampling event to ensure proper sample collection, handling, storage and transfer, and record any site issues.

Wastewater virus nucleic acid was concentrated and extracted using Nanotrap Magnetic Virus Particles (Ceres Nanosciences, Manassa, VA, USA) following manufacturer’s protocols. Prior to concentration, wastewater samples were spiked with 6000 genome copies per microliter (GC/µL) murine hepatitis virus (MHV) stock solution. Thermo Scientific Invitrogen MagMAX™ Microbiome Ultra Nucleic Acid Isolation Kit was used following the recommended protocol on KingFisher™ Flex system for nucleic acid extraction. The extracted nucleic acid was analyzed for quantifying SARS-CoV-2 N2 and E genes, MHV using multiplex RT-ddPCR using PREvalence ddPCR SARS-CoV-2 Wastewater Quantification Kit (Bio-Rad, USA) following the manufacturer recommended protocols. PMMoV was used as a human fecal marker and was quantified by RT-ddPCR using diluted extracted nucleic acid. The QX Manager Software Standard Edition paired with the PREvalence Wastewater Automated Analysis Application was used to manually validate all positive calls and analyze/threshold droplet clusters. The limit of detection for N2 gene, E gene, and MHV are 5, 2, and 30 GC/µL, respectively. A detailed description of the manufacturer’s recommended protocols were provided in Pappu et al. [4].

To ensure quality control and quality assurance through the sample concentration, nucleic acid extraction and ddPCR, SARS-CoV-2 and MHV were run in triplicates for each sample. MHV was run as both process control and positive control. All PMMoV assays were run in duplicate for each sample. To ensure validity of results, positive and negative controls were included on each ddPCR plate. Master mix ratios and thermal cycling conditions provided by the Prevalence Kit were followed accordingly.

A geographic information system (GIS) based dashboard was built for spatio-temporal visualization of infection hotspots across monitored communities. AutoCAD and ArcMap were used to build spatial files identifying all sewer manhole locations and residential housing communities served by the manholes. The manhole sites and their upstream building were annotated with unique IDs in the spatial file developed using ArcMap. Both the manhole sites shapefile and the building shapefile were merged using the IDs. The combined shapefile was transformed into a coordinate reference system using georeferencing method. The average wastewater SARS-CoV-2 N2 gene concentrations data from each sewer manhole was transformed into ArcMap compatible format. The transformed wastewater data was merged with the georeferenced spatial file in Tableau for visualizing infection hotspot clusters. To ensure proper data assurance through sample collection, data compilation and data visualization, samples were thoroughly annotated and labelled with their site IDs and dates. Additional details regarding the dashboard development were provided in Pappu et al. [4].

All statistical analysis was performed using RStudio®. Spearman correlation analysis was performed using function corr() to assess the association between wastewater SARS-CoV-2 N2 gene concentration and clinical testing data both with and without normalization. The 3-day and 7-day rolling averages of clinical cases were estimated by dividing the sum of values within the selected period (3 days or 7 days) by the number of values in the period (3 or 7). For quantitative statistical trend analysis, segmented linear regression was conducted on the LOESS fit log transformed wastewater SARS-CoV-2 N2 data normalized by population size to estimate breakpoints, their dates, and phases of major trend changes. R package zoo [5] and function rollmean() were used to estimate the 3-day and 7-day rolling averages of clinical cases. R package segmented [2] was used to estimate breakpoints. R package spatialEco [1] and linear model lm() function were used to estimate the direction and magnitude of trend change using Mann Kendall tau (MK) and daily percent change (PC), respectively. For estimating PC values, the slope from lm() function was extracted and applied to the daily percent change equation (Eq. (1)) by United States Center for Disease Control’s National Wastewater Surveillance System [3].

(1) $$PC=(10^{Slope}-1)*100$$

## Limitations

During the study, sampling failures events occurred most frequently at communities with population below 700. Failures occurred commonly from low flow velocities, sporadic wastewater flows, and clogging of intake port by debris in wastewater. Community A, which represents the smallest community among the monitored sites with population below 500, had the highest sampling failure events. Community A dataset was excluded from data analysis because of large data gaps from failed sampling events. This limited our ability to make accurate trend predictions for Community A.

## Ethics Statement

The authors of this paper have read and agree with the ethical statements of this journal. This study does not involve human subjects or animal subjects. Also, the data provided in this article does not contain any data from social media platforms.

## CRediT Author Statement

**Aiswarya Rani Pappu:** Conceptualization; Data curation; Formal analysis; Investigation; Methodology; Software; Visualization; Roles/Writing – original draft; Writing – review & editing. **Ashley Green:** Data curation; Methodology; Writing – review & editing. **Melanie Oakes:** Data curation; Methodology; Resources. **Sunny Jiang:** Conceptualization; Funding acquisition; Investigation; Project administration; Supervision; Validation; Writing – review & editing.

## Data Availability

Mendeley DataWastewater surveillance data (Original data).

## References

[1] Evans J.S., Ram K. (2021).

[2] Muggeo V.M., Muggeo M.V.M. (2017). Package ‘segmented’. Biometrika.

[3] National Wastewater Surveillance System (NWSS) (2022). https://www.cdc.gov/nwss/reporting.html.

[4] Pappu A.R., Green A., Oakes M., Jiang S. (2025). Tracking COVID-19 trends in communities with low population by wastewater-based surveillance. Sci. Total Environ..

[5] A. Zeileis, G. Grothendieck, J.A. Ryan, F. Andrews and M.A. Zeileis, 2014. Package ‘zoo’. R package version, pp.1–7.

[6] A. Green, A.R. Pappu, M. Oakes, S. Sandmeyer, M. Hileman, S. Jiang, Variabilities in N2 and E Gene Concentrations in a SARS-CoV-2 Wastewater Multiplex Assay. personal communication. 10.3390/microorganisms13081862 PMC12388642 40871365

